# Patient outcomes and experiences of an acupuncture and self-care service for persistent low back pain in the NHS: a mixed methods approach

**DOI:** 10.1186/1472-6882-13-300

**Published:** 2013-11-01

**Authors:** Anna Cheshire, Marie Polley, David Peters, Damien Ridge

**Affiliations:** 1 Faculty of Science and Technology, University of Westminster, 115 New Cavendish Street, W1W 6UW, London, UK, England; 2 Polyclinic, University of Westminster, 115 New Cavendish Street, W1W 6UW, London, UK, England

**Keywords:** Beating back pain, Self-management, Acupuncture, Information, Evaluation

## Abstract

**Background:**

Supported self-management, acupuncture and information can help reduce the symptoms of low back pain. These approaches are currently recommended by NICE guidance as treatment options for patients with persistent low back pain. However, there has been no previous evaluation of a service providing them together for this common problem. The purpose of this service evaluation was to report patient outcomes and experiences of the Beating Back Pain Service (BBPS), a pilot service based in a primary and community care setting, delivering acupuncture, self-management and information to patients with chronic low back pain.

**Methods:**

Patients completed a questionnaire at three time points: pre-BBPS, immediately post-BBPS and three months post-BBPS. Outcome measures included the Bournemouth Questionnaire (measuring musculoskeletal, MSK, problems), EuroQoL-5D (measuring quality of life), Pain and Self-efficacy Questionnaire, and additional questions on medication use, physical activity, understanding of pain and positive well-being. Additionally, the STarT Back (measuring risk of developing chronic pain) was collected at BBPS information sessions. Non-parametric tests were used to evaluate pre- and post- variables. Questionnaires also collected qualitative data (open-text responses) regarding patient views and experiences of the BBPS, which were analysed using thematic analysis.

**Results:**

80 (out of 108) patients who attended the initial BBPS information session agreed to participate in the service evaluation (mean age 47 years, 65% female). 65 patients attended subsequent BBPS acupuncture and/or self-management sessions and were asked to complete post-treatment questionnaires; complete datasets were available for 61 patients.

There were statistically significant improvements over time for pain (p <0.0001), quality of life (p = 0.006), understanding of pain (p <0.001), physical activity (p = 0.047) and relaxation (p = 0.012). Post-hoc analysis revealed that scores improved between baseline and post-treatment, these improvements were maintained at 3-month follow-up (except relaxation). Patients receiving a combination of acupuncture and self-management sessions produced the most positive results. Patient satisfaction with the BBPS was high.

**Conclusions:**

The BBPS provided a MSK pain management service that many patients found effective and valuable. Combining self-management with acupuncture was found to be particularly effective, although further consideration is required regarding how best to engage patients in self-management.

## Background

One third of the UK population is affected by low back pain each year and around 20% of those affected (that is, 1 in 15 of the population) will consult their GP about their pain [1]. For 62% of people with low back pain the problem endures for over 12 months [2]. In the UK back pain has been estimated to cost the economy £12.3 billion per year [3] and places a heavy burden on primary care services [4]. At the same time, treatment for musculoskeletal (MSK) problems is perceived by GPs and other health professionals as an ‘effectiveness gap’ within the NHS [5,6]. Furthermore, the Chief Medical Officer’s 2008 report recommended that much more needs to be done to improve outcomes for patients with MSK pain, arguing that patient-centred services are essential. Current systems and infrastructure, however, are inadequate to meet both patient needs and level of demand [3]. Taken together these finding suggest that more needs to be done to meet the needs of patients with low back pain and that it is cost-effective to prevent back pain becoming chronic.

NICE (National Institute for Health and Care Excellence) guidelines recommend that self-management, acupuncture and information should be provided for patients with persistent low back pain [1], however, there has been no evaluation of an NHS service which makes these all available for this common problem. Yet there is good evidence for the potential benefits of acupuncture, self-management and information as treatment options for low back pain. Evidence from randomised controlled trials (RCTs), meta-analyses and a Cochrane review demonstrates that acupuncture can be useful in reducing low back (including chronic) pain e.g. [7-13] compared to control groups. Acupuncture can also be useful when combined with other interventions including education and behaviour modification [13-15]. Randomised controlled trials, reviews and meta-analysis have also shown that self-management courses can be clinically effective in terms of improving pain (including low back pain), compared to control groups (controls include usual care, inpatient or outpatient non-multidisciplinary treatments, wait-list controls or alternative treatments) [16-19]. Systematic reviews have shown that providing chronic low back pain patients with advice and information can improve pain and functionality [20,21], but more studies are needed in this area [20,22]. In addition, providing patients with information regarding the effectiveness of self-management and their pain may support patient self-management [23].

Acupuncture and self-management may also have wider benefits than pain reduction. Patient outcomes and experience data suggest that acupuncture may also improve patients’ quality of life and well-being, reduce medication use, and improve coping/self-management [24-27]. Additionally, high levels of GP and patient satisfaction are often reported with services that include acupuncture [24-27]. Self-management has also been shown to have wider benefits, such as improvements in self-efficacy and cognitive coping, better energy levels and emotional well-being, reduced fatigue, and increased daily functioning [16-19].

It is important to consider how the NHS can best translate such research findings to provide effective treatments that are also acceptable to patients with low back pain. Yet, no studies have assessed the effectiveness (i.e. how well interventions work in the real world) of a service providing acupuncture, self-management and information for chronic low back pain on the NHS. Evaluation is crucial to determine how to properly deliver complex treatment pathways to achieve the best clinical outcomes and acceptability. The current movement within the NHS towards a more user-centred service where the Government is keen that any modernisation of the NHS involves putting patients “at the centre of everything the NHS does” [28]. Thus, it is particularly important to understand patient perspectives and experiences of services, not just outcomes. The current paper reports on a service evaluation which uses mixed methods [29] to report on both patient outcomes and experiences of the Beating Back Pain Service (BBPS), a pilot service provided in a primary and community care setting, delivering acupuncture, self-management and information to patients with chronic low back pain.

## Methods

### The Beating Back Pain Service

The BBPS was provided within a Primary Care Trust (PCT) between October 2010 and December 2011^1^ and delivered within a primary and community care setting. It aimed to provide early intervention for low back pain patients in order to reduce the use of chronic pain management services and referrals to secondary care. The BBPS accepted referrals of patients from GPs, and NHS physiotherapists and osteopaths. Inclusion criteria included: diagnosis of non-specific low back pain of more than six week duration, aged over 18 years old, and patient initially willing to participate in the service and evaluation. Exclusion criteria included: presence of red flags^2^, inability to communicate in English (no money was available for translation), mental health problems, and substance abuse. On referral to the BBPS all patients initially attended a group session that provided information on pain and how to manage it. Based upon their risk of developing chronic pain (measured by STarT Back as described below) patients could then elect to receive an individualised combination of acupuncture, self-management groups and using the BBPS pack (booklet and CD with information and exercises for mobility and strength to manage back pain, provided to every patient attending information sessions). Patients identified as most at risk for developing persistent symptoms were encouraged to attend acupuncture and self-management sessions, rather than just acupuncture and/or BBPS pack. This new service design was informed by current guidelines which recommend the provision of acupuncture, self-management and information for persistent low back pain [1] and the integration of risk factors (such as those measured by the STarT Back) with back pain management [30].

### Information sessions

Information sessions were group sessions (for up to 12 patients) initially provided to all BBPS patients as the single point of entry to the Service. They were delivered by two healthcare professionals: a qualified GP and musculoskeletal specialist also trained in osteopathy and acupuncture, and an occupational therapist also trained in psychotherapy. Sessions lasted two hours and aimed to improve participants’ understanding of how the cycle of back pain and tension operates, the effects of mood and stress, the importance of movement and exercise and, in the light of this model of back pain, how to manage pain more appropriately. They also encouraged patients to share their experiences of back pain and their ways of coping with it. During sessions patients and facilitators decided which interventions were likely to be most helpful for patients using the STarT Back tool [31] - a questionnaire which helps to identify patients most at risk of developing persistent symptoms.

### Acupuncture

Patients referred to acupuncture received up to six weekly sessions (lasting 30 minutes, 45 minutes for first session) of individualised Traditional Chinese Medicine (TCM) acupuncture treatment. Acupuncture sessions were delivered by a senior acupuncturist (17 years post qualification experience) trained in TCM, with experience of working in the NHS and registered with the British Acupuncture Council. During the first session a full case history was taken along with traditional pulse and tongue diagnosis. From these, a treatment plan was developed, which could be adjusted each week depending on the patient’s response to treatment. Patients received treatment primarily for their low back pain.

### Self-management groups

The self-management course comprised group sessions structured to provide on-going drop in support, in order to meet patient needs flexibly. Sessions aimed to provide patients with the knowledge, skills and on-going support to manage their back pain and address psychosocial obstacles to recovery. Topics covered included breaking the pain-tension cycle, managing pain and stress, pacing, goal setting, staying active and relaxation, and incorporated elements of mindfulness and cognitive behavioural therapy (CBT) [1,32,33]. Sessions included explanation time, activity time and group discussion/support. The course was designed and delivered by a qualified occupational therapist and psychotherapist who has extensive experience in stress management and emotional resilience, and working with a wide range of clients. She is a full member of the Institute of Stress Management. She was supported in the delivery of the course by another qualified psychotherapist and body worker, who was able to provide information on the physiology of back pain on the course.

### The service evaluation

In order to evaluate patient outcomes and experiences of the BBPS, data were collected using patient questionnaires and the STarT Back tool (see below). Ethical approval for the evaluation was obtained from the University of Westminster Ethics Committee (reference number 09/10/41). The NHS confirmed the study to be an evaluation, thus NHS ethics was not required. Informed written consent was collected from all participants. The evaluation was conducted by the authors AC, MP and DR, all of whom are independent researchers and were not part of the BBPS Team in any way.

### Patient questionnaires

All patients using the BBPS were invited to complete questionnaires at key time points. Questionnaires were used to collect quantitative (and some qualitative) data from patients at three time points: immediately pre-BBPS, on completion of the BBPS and 3 months after completion of the BBPS. Patients were provided with a questionnaire pack (containing all three questionnaires, addressed pre-paid envelopes for returning questionnaires, and the patient information sheet and consent form) by a researcher who attended BBPS information sessions to explain the research. Patients completed their pre-treatment questionnaire at the BBPS information sessions and were sent texts or had telephone call prompts when it was time to return their post-treatment and 3-month follow-up questionnaires. Identical copies of the questionnaires were also available to be completed online, according to patient preference. The following data were collected:

MSK pain, which was measured using the Bournemouth Questionnaire (BQ) core items [34]. The BQ is a pre-validated questionnaire developed specifically for patients with MSK pain and has been shown to be reliable, valid and responsive to clinical change e.g. [34]. The BQ incorporates dimensions of the biopsychosocial model for MSK pain including levels of pain, interference with everyday tasks and social activities, anxiety, depression, the extent to which work affects their condition and coping ability. It comprises seven items scored from 0 to 10 which can then be summed to provide a total score ranging from 0 to 70. Higher scores indicate increased MSK problems.

Quality of Life (QoL), which was measured using the EuroQol-5D (EQ-5D) [35] a pre-validated, widely used, generic measure of health-related quality of life. It is quick and easy to complete and has been shown to be valid and reliable [36,37]. The first part comprises five items (measuring mobility, self-care, usual activities, pain and anxiety/depression) which are graded on three levels according to severity. Using the established algorithms for the UK these items were translated directly into index scores, ranging from -0.59 (worst possible health state) to 1 (best possible state). The second part is a visual analogue scale (VAS) measuring overall health, anchored 0 (worst possible health state) to 100 (best possible health state).

Self-efficacy for managing pain, which was measured using the Pain and Self-efficacy Questionnaire (PSEQ) [38]. The PSEQ is a pre-validated questionnaire measuring patient beliefs regarding their ability to perform activities whilst in pain. The scale has been shown to be valid and reliable among patients with low back pain [38], and to predict pain-related behaviour [39]. The scale comprises 10 items scored from 0 to 6 which are summed to provide a total score ranging from 0 to 60, with higher scores indicating stronger self-efficacy beliefs.

Positive well-being, which was measured using 5 different questions asking participants to rate their understanding of their pain, positivity, hope, ability to face up to health problems and relaxation, on a scale of 0 (strongly disagree) and 10 (strongly agree).

Participants were also asked if they were using analgesics, about areas where they experienced pain and work status. They were also asked to rate their physical activity levels on a scale of 0 (not at all active) to 10 (extremely active). Demographic data (age, gender, ethnicity) were collected in the pre-treatment questionnaire only.

Qualitative data were collected via open-ended questions (providing free text boxes for answers) at the end of questionnaires. The pre-treatment questionnaire asked patients what they had learned from the information session. The post-treatment questionnaire asked patients about any benefits they had got from the acupuncture/self-management course, improvements that could be made to the service, if there was anything else in their life that may be affecting their health, or any other comments they would like to make about the Service.

### The STarT Back Questionnaire

The STarT Back Questionnaire [31] was designed to identify patients most at risk of developing persistent low back pain, in order to aid decision making and target treatment more effectively. It comprises nine questions which are then used to split patients into low, medium and high risk of poor outcome. It has established reliability and validity [31,40] and its use has been shown to achieve greater health benefits for patients at a lower cost to the NHS [41]. The STarT Back Questionnaire was completed by BBPS patients in information sessions, to help the BBPS Team guide participants towards the most appropriate BBPS interventions.

### Data analysis

To assess whether this service design had a beneficial effect for the patients, quantitative data were analysed using SPSS version 19. Statistical significance was set at the 5% level. To ensure a conservative analysis, non-parametric tests [42,43] (Friedman, Mann Whitney-U, Wilcoxon Signed Rank, Kruskal-Wallis, McNemar and Chi-square as appropriate) were used to compare the differences between those who did and did not return questionnaires on baseline variables. Non-parametric tests were further used to compare pre-, post- and follow-up treatment variables including the BQ, EQ-5D, PSEQ, positive well-being, physical activity, analgesic use and current work status. Percentage of participants experiencing a clinically significant improvement was determined by calculating the effect size for the BQ (raw change score divided by the standard deviation of the baseline scores). An effect size of 0.5 has been found to represent a clinically significant change for the BQ [44].

To assess the value of providing self-management and acupuncture together, data were examined for differences between patients who attended acupuncture and self-management sessions compared with those who attended acupuncture only. Change scores were calculated for all study variables and compared using Mann Whitney-U tests for pre- and post-treatment, and pre-treatment and follow-up.

In order to establish if the BBPS was meeting its aim of providing an early intervention to prevent the need for patients at high risk of developing persistent symptoms using chronic pain management services, we compared BQ change scores (between baseline and 3-month follow-up) for patients categorised as low, medium and high risk of poor outcome (as identified by the STarT Back Questionnaire).

Qualitative data collected from open ended questions on the questionnaires were analysed using thematic analysis [45]. Analysis aimed to explore patient experiences, opinions and acceptability of the Service. The first author (AC) immersed herself in the data highlighting key sections of text and words to develop an initial list of themes/codes. This list was then debated with the fourth author (DR) to arrive at a final coding list. The first author inputted and coded all the data in the qualitative data analysis software environment, NVivo [46]. Typical quotes are used to illustrate findings. Participant identification numbers are used to protect participant anonymity.

## Results

The results are presented in three sections. Firstly, patient characteristics and response rates are presented. The second section examines patient outcomes using quantitative data from patient questionnaires and the STarT Back tool. The final section reports on patient experiences and views of the BBPS using qualitative data from questionnaires.

### Participant characteristics and response rates

All patients who attended an information session were invited to take part in the evaluation. Eighty patients chose to participate, 74% of the total number of patients attending information sessions. Participants’ characteristics are reported in Table 1. Fifteen (18.8%) patients attended an information session only, 47 (58.8%) received acupuncture only, 1 (1.3%) person attend self-management sessions only, and 17 (21.3%) attended self-management and acupuncture sessions. Those receiving acupuncture attended an average of 5.2 (range: 1 to 12) sessions. Those receiving self-management attended an average of 9.3 (range: 1 to 31) sessions.

**Table 1:** Participant characteristics

**Characteristics**	**Figures**
Gender	mean 35% Males; 65% females
Age	mean 47 years old, range 18 - 83
Ethnicity	
White	39%
Afro-Caribbean/African	14%
Asian	9%
Arabic	9%
Mixed	6%
White-European	6%
Other	7%
Missing	10%
Mean time current painful episode has lasted	78 wks, range 0.5 – 1092
Have previously experienced a complaint similar current episode	74%
Majority have pain in:	
Lower back	91%
Leg or knee	53%
Shoulders	40%
Neck	39%
Pain in more than one area of the body	80%
Pain in more than two areas of the body	46%
Taking pain medication	78%
Reporting anxiety and depression	
Moderate	54%
Extreme	16%

Of the 80 patients attending an information session and participating in the evaluation, 65 attended acupuncture and/or self-management sessions and were asked to complete post-treatment questionnaires, 61 (93.8%) completed both their post-treatment and 3-month follow-up questionnaires. No statistically significant differences were found on demographic or study variables between responders and non-responders. All statistical analyses of the patient outcome data are based upon the 61 completed data sets.

### Patient outcomes

#### Changes in MSK pain

For the primary outcome measure of MSK pain (BQ), comparisons revealed a statistically significant improvement over time (pre-treatment, post-treatment and 3-month follow-up) in MSK problems for BQ total score (p <0.0001) and four out of seven subscales: pain (p <0.0001), interference with daily activities (p = 0.023), interference with social routine (p <0.0001) and anxiety (p = 0.024). There was a trend towards an improvement for the effect on work subscale (p = 0.075). No statistically significant differences were found for 2 of the subscales: depression (p = 0.334) and coping (p = 0.412). Post-hoc comparisons revealed that the above improvements in scores occurred between baseline and post-treatment and were maintained at 3-month follow-up (p ≤ 0.05) for BQ total score and the subscales pain, interference with daily activities and anxiety; there was a trend towards maintained improvement for the interference with social routine subscale, see Table 2.

**Table 2:** BQ total and sub-scale scores over time, pre-treatment, post treatment and 3-month follow-up (n = 61)

	**Pre-treatment Median (interquartile range)**		**Post-treatment Median (interquartile range)**		**3-month FU Median (interquartile range)**		**p-value†**
**BQ total score (range 0-70 ↑ = worse)**	**46.0**	**(35.0-53.0)**	36.0*	**(23.5-46.0)**	40.0*	**(25.5-51.0)**	**<0.0001**
BQ subscales (range 0-10 ↑ = worse)							
Pain	8.0	(6.0-8.0)	6.0*	(4.0-8.0)	6.0*	(4.5-7.8)	<0.0001
Interference with activities	6.0	(4.8-8.0)	5.0*	(3.0-7.0)	5.0*	(4.0-7.0)	0.023
Interference with social	6.0	(4.0-8.0)	4.0*	(3.0-7.0)	6.0^a^	(2.3-8.0)	<0.0001
Anxiety	7.0	(5.0-8.0)	5.0*	(3.0-7.0)	6.0*	(2.0-7.0)	0.024
Depression	6.0	(4.0-8.0)	5.0*	(2.5-7.0)	5.0	(3.0-7.0)	0.334
Effect on work	7.0	(4.0-8.0)	5.0*	(3.0-7.0)	6.0	(4.0-8.0)	0.075
Coping	5.0	(4.0-7.0)	5.0^a^	(3.0-6.0)	5.0	(4.0-7.0)	0.412

*Significantly different from pre-treatment (p ≤ 0.05).

^a^ Approaching significantly different from pre-treatment (p = 0.051 to 0.099).

†P-value refers to Friedman Tests which measure change in scores over 3 points in time (pre-treatment, post treatment and 3-month follow-up).

↑Refers to higher scores.

Applying the threshold of 0.5 for effect size [44], 24 (39.3%), 95% CI [27.1%, 51.6%] participants experienced a clinically significant reduction in their MSK pain immediately post-treatment and 23 (37.7%), 95% CI [25.5%, 49.9%] participants experienced a clinically significant reduction in their MSK pain at 3-month follow-up. 18 (29.5%) participants experienced a clinically significant reduction in pain at both post-treatment and 3-month follow-up, 6 (9.8%) participants experienced a clinically significant reduction at post-treatment only, and 5 (8.2%) participants experienced a clinically significant reduction at follow-up only.

#### The STarT Back Questionnaire

STarT Back scores showed that 23 (37.7%) patients were at high risk of developing chronic low back pain, 19 (31.1%) were at medium risk and 9 (14.8%) were at low risk. Data were unavailable for 10 (16.4%) patients. In order to see if the STarT Back questionnaire was a useful way to inform the triaging of patients, we first sought to establish if the STarT Back differentiated patients on severity of their condition. Total BQ baseline scores were compared for patients identified as low, medium or high risk of poor outcome (as identified using the STarT Back): The Kruskal-Wallis test showed a statistically significant difference (p <0.0001). Post-hoc analysis revealed that there were statistically significant differences between low and medium (p = 0.0003), medium and high (p = 0.035) and low and high risk groups (p <0.0001). Confirming the ability of the STarT Back to triage into ‘at risk’ groups. We then sought to establish if treatment decisions based on STarT Back scores effected patient outcomes (without treatment low risk patients would be expected to improve more than higher risk patients). No statistically significant difference was found on BQ change scores at 3-month follow-up between patients identified as low, medium or high risk of poor outcome (p = 0.382). This finding suggests patients are experiencing an improvement in their pain (or not), regardless of their risk status.

#### Changes in patient quality of Life

For health-related QoL (EQ-5D index and EQ-5D VAS), comparisons revealed a statistically significant improvement over time (pre-treatment, post-treatment and 3-month follow-up) for QoL EQ-5D index (p = 0.006). Post-hoc analysis revealed that improvements in scores occurred between baseline and post-treatment and were maintained at 3-month follow-up. There was a trend towards an improvement in EQ-5D VAS (p = 0.074), see Table 3.

**Table 3:** Study variable scores over time, pre-treatment, post treatment and 3-month follow-up (n = 61)

	**Pre-treatment Median (interquartile range)**		**Post-treatment Median (interquartile range)**		**3-month FU Median (interquartile range)**		**p-value†**
EQ-5D - index (range -0.-.59-1 ↑ = worse)	0.19	(-0.02-0.69)	0.62*	(0.32-0.73)	0.62*	(0.08-0.74)	0.006
EQ-5D – VAS (range 0-100 ↑ = better)	60.0	(38.3-70.0)	61.0*	(40.0-75.0)	60.0	(40.0-80.0)	0.074
PSEQ (range 0-60 ↑ = better)	34.0	(15.2-44.0)	39.0*	(22.6-47.0)	37.0	(19.5-49.0)	0.286
Physical activity (range 0-10 ↑ = better)	5.0	(3.0-7.0)	6.0*	(4.0-7.0)	6.0*	(3.0-7.0)	0.047
Positive well-being scales (range 0-10 ↑ = better)							
Understanding of pain	5.0	(3.0-7.0)	7.0*	(4.0-8.0)	6.0*	(3.5-8.0)	<0.0001
Positivity	6.0	(3.0-8.0)	6.0*	(4.0-8.25)	6.0	(4.0-8.0)	0.265
Hope	6.0	(4.3-8.0)	6.0	(4.0-8.0)	6.0	(4.0-8.0)	0.207
Ability to face up to health problems	6.0	(5.0-8.0)	7.0*	(5.0-8.0)	6.0	(4.0-8.0)	0.779
Relaxation	5.0	(3.0-6.0)	6.0*	(4.0-8.0)	5.0	(3.0-7.0)	0.012

*Significantly different from pre-treatment (p ≤ 0.05).

†p-value refers to Friedman Tests which measure change in scores over 3 points in time (pre-treatment, post treatment and 3-month follow-up).

↑Refers to higher scores.

#### Changes in patient understanding of pain, physical activity and positive well-being

Comparisons revealed a statistically significant improvement over time (pre-treatment, post-treatment and 3-month follow-up) in understanding of pain (p ≤ 0.001), physical activity (p = 0.047) and ability to relax (p = 0.012). Post hoc comparisons revealed that statistically significant improvements in scores occurred between baseline and post-treatment and were maintained at 3-month follow-up for understanding of pain (p = 0.008) and physical activity (p = 0.042), but not relaxation (p = 0.160). There was no change in ability to self-manage PSEQ (p = 0.286), positivity (p = 0.265), hope (p = 0.207), or ability to face up to health problems (p = 0.779), see Table 3.

#### Changes in patient medication use and work status

There was no change in medication use (p = 0.920) or current work status (p = 0.368).

#### Understanding the benefits of providing self-management with acupuncture

To establish if attending self-management training in addition to having acupuncture was beneficial for the 17 patients who received it, data for this group were analysed. Results showed that patients who attended acupuncture and self-management sessions improved more than patients who attended acupuncture only; post-treatment there was a statistically significant difference for MSK pain (p = 0.022) and a trend towards improvement in health-related QoL (EQ-5D index) (p = 0.057), these differences were still evident at 3-month follow-up (p = 0.047 and p = 0.057 respectively). In addition, at 3-month follow-up (but not post-treatment), there were statistically significant improvements for hope (p = 0.041) and ability to face up to health problems (p = 0.050), and trends towards improved positivity (p = 0.063) and ability to self-manage PSEQ (p = 0.061), for those who attended both (see Table 4). The suggestion here is that improvements in these areas may take time to develop and are promoted by learning self-management strategies in addition to acupuncture. Qualitative data regarding self-management sessions are presented in the ‘changes to the patient’s condition’ section below.

**Table 4:** Change scores for patients receiving acupuncture and self-management compared with patients receiving acupuncture only

	**Post-treatment change scores**		**p-value**	**Follow-up change scores**		**p-value**
	**Ac**	**Ac + SM**		**Ac**	**Ac + SM**	
BQ total score	-4.6	-16.6	0.022	-2.1	-13.3	0.047
EQ-5D - index	0.15	0.36	0.057	0.10	0.31	0.057
EQ-5D – VAS	7.1	4.5	0.845	0.7	6.8	0.334
PSEQ	2.5	6.9	0.261	0.6	7.6	0.061
Physical activity	0.7	0.4	0.749	0.5	0.8	0.911
Understanding of pain	1.3	2.2	0.256	0.9	1.7	0.534
Positivity	0.5	1.8	0.158	-0.2	1.8	0.063
Hope	0.5	1.2	0.188	-0.7	0.8	0.041
Ability to face up to health problems	0.0	0.2	0.833	-0.8	0.6	0.050
Relaxation	1.3	0.9	0.492	0.1	1.3	0.107

### Patient experiences and views of the BBPS

This section first explores patients views and experiences of the BBPs information session using predominantly qualitative data taken from the baseline questionnaire, it then moves on to explore patients overall experiences of the BBPS service using qualitative data from the post-treatment questionnaire. Findings are presented around the themes shown in Table 5.

**Table 5:** Themes for Patient experiences and views of the BBPS

**Data**	**Themes**
Experiences of the BBPS information session	Outcomes of attending the information session
	The group format
	Receiving support
	Content of sessions
Overall experience of the BBPS (collected post-treatment)	Changes to the patient’s condition
	Suggested improvements for the Service
	Attributes of BBPS practitioners

#### The BBPS information session

**Figure 1:**
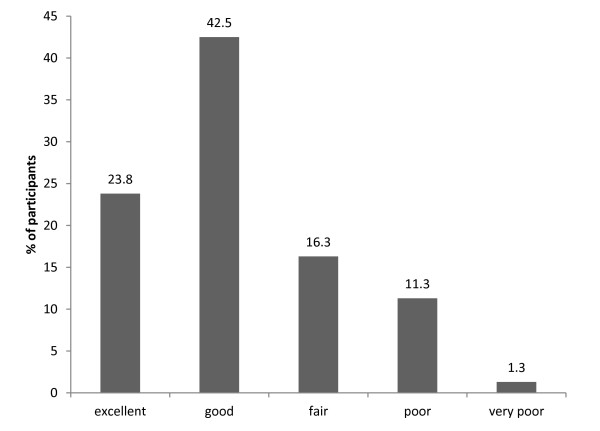
Patient ratings for the information session.

Patients provided an overall rating of the information session; the majority of patients rated it as ‘good’ or ‘excellent’ (see Figure 1).

##### Outcomes of attending the information session

Patients described information sessions as “informative” and reported a range of learning as a result of attending. Reported outcomes of attending information sessions included:

“Before I came to this meeting I didn’t understand much about my pain, but now I do. I think it is very good that the PCT is doing this ‘Beating Back Pain’. Very helpful.” P35

• Increased knowledge and understanding of chronic pain such as how the spine works, what causes pain and what to do to help alleviate it.

• Understanding of the importance of keeping active in managing pain –that is was important to do exercises and stretching and that this would not make the pain worse.

• Increased understanding of the body-mind connection in pain and the importance of relaxation, breathing and positivity for pain management.

• Increased knowledge of the kinds of treatments that might help pain (e.g. acupuncture and osteopathy).

• Learning other pain management strategies and tips, for example how to get out of bed in the morning or using heat and cold.

• Only a small number (n = 3) of patients felt that they had learnt little from attending the session.

##### The group format

Many patients reported enjoying the group format. They generally liked meeting others with similar problems to their own - people who understood what it was like to live with pain. They enjoyed sharing their experiences and listening to the experiences of others. However, not all patients enjoyed the group format, and said that discussing pain with others was not for them.

“Nice to hear other people have similar problems and understand. Can be difficult when others can’t see your injury to sympathize.” P16

##### Receiving support

Some patients reported that after the session they felt encouraged and supported. They felt more hopeful about their future, and grateful that people had taken time to listen to their experiences. Some reported they wished they had received this kind of information and support years ago.

“Pain clinic has been very helpful for me I wished I could get this help years ago. I feel I have a support for all my pains. People who understand exactly what I’m going through.” P32

##### Content of sessions

Although many patients reported they liked the content of information sessions, a few said that they had been expecting (and wanting) something more diagnosis-based. A small number of patients would have liked more information on what treatments were available to them, feeling that this part of the information session was mostly directed towards acupuncture.

#### Overall experiences of the BBPS

##### Changes to the patient’s condition

Patients’ overall experiences of the BBPS complemented findings from the quantitative data. When asked about changes to their condition since attending BBPS sessions, many patients described improvements in their pain. These reports ranged from complete to temporary pain relief. Some patients reported that this relief had led to improved mobility and relaxation, and reduced muscle tightness. Some patients reported psychological benefits as a result of treatment such as feeing more in control, confident, positive, hopeful and ‘mentally stronger’. Patients especially described feeling better able to manage their pain. Some patients reported that the self-management group had been particularly useful for providing them with support and teaching them how to manage their pain. Others reported increased knowledge regarding their condition.

“Learnt how to manage pain useful CD/leaflet/group to encourage and learn various routines both physical and mental. Changed my approach to coming positive, energised – a can do practice with planning and pacing.” P33

“Pain relief, sense of well-being, mentally stronger as I felt I was tacking the problem. Fantastic advice from [acupuncturist].” P19

Some participants said that treatment had not helped their pain and one reported a temporary worsening of their condition after treatment. One patient reported sometimes feeling tired and depressed after acupuncture treatment.

“For my problem I didn’t notice a big benefit.” P79

##### Suggested improvements for the service

When asked how acupuncture and self-management sessions could be improved, the overwhelming number of suggestions related to expanding and extending the service. Patients wanted more acupuncture sessions of longer duration, others suggested maintenance sessions would be appropriate. Some patients wanted to see the whole service more widely available on the NHS and one person suggested the service could be extended to people with all types of pain. Three patients reported that they would have liked more flexible times / locations for the self-management course. One patient explained how some of the self-management group were hoping to continue meeting once sessions had finished so that they could continue to share experiences and support one another.

“Happy with the quality, could have done with more sessions.” P31

“Six sessions are not enough to treat someone who has severe back pain. I also think there is a need of maintenance as per acupuncture principles.” P13

“[Make it] more readily available on the NHS.” P14

Other changes to the Service suggested by patients included the provision of written information such as online self-management information or a typed sheet explaining why acupuncture should work. One patient suggested having more information on the self-management course related to posture and what is good and bad for the back, this information was subsequently added to the self-management sessions. One patient suggested an online booking facility; another would have liked to have seen the BBPS more linked with osteopathy and chiropractor courses.

“Both courses should be linked with osteopathy and chiropractor courses.” P22

“Maybe a printed out sheet of the treatment given with explanation of why it should work would be helpful.” P80

##### Attributes of BBPS practitioners

Many participants praised the practitioners that delivered the BBPS; they had found practitioners professional, knowledgeable and efficient. In particular, what was prominent in the analysis was the praise practitioners received for their humanistic qualities, including kindness, understanding, empathy, encouraging and caring.

“I felt that I was listened to when I was describing what was going on and they even took note and interest in my other medical problems. Seemed more understanding and compassionate than any consultation I have had under the NHS.” P61

“The people who run the course are very professional, caring and very friendly. They give much support and help to us all. [Acupuncturist] is wonderful, very professional and efficient.” P35

## Discussion

This service evaluation reported on patient outcomes and experiences of the BBPS, a pilot service delivering acupuncture, self-management and information to patients with chronic low back pain. This pilot service was delivered in a primary and community care setting and helped to implement NICE (National Institute for Health and Care Excellence) guidance for persistent low back pain locally, by working with local GPs and health professionals [1]. Findings showed that patients using the BBPS experienced improvements in their pain, quality of life, understanding of their pain, levels of physical activity and levels of relaxation, which continued for 3 months after they finished treatment (with the exception of relaxation). These findings demonstrate that this type of service can achieve results in line with other research suggesting that acupuncture and self-management can help with the reduction of low back pain e.g. [7-13,16-19], as well as having wider benefits such as improved quality of life, psychological well-being and self-efficacy [16-19,24-27]. Our findings also suggest that providing self-management *with* acupuncture for patients most at risk of developing chronic pain worked best, particularly 3 months post intervention. A short course of acupuncture may relieve patients back pain, but if causal factors linked to pain (e.g. sedentary lifestyle, stress, maladaptive coping strategies) are not rectified relapse may occur. Thus, our findings show that self-management training may work synergistically with acupuncture.

BBPS patient treatment recommendations (exercise at home, acupuncture and/or self-management) were based on the patient’s risk of developing chronic pain, which was ascertained using the STarT Back questionnaire completed by patients at BBPS information sessions. The importance of tailoring back pain treatment with individuals’ prognostic indictors has been highlighted by researchers and clinical guidelines [47]. A recent study demonstrated the potential effectiveness of using the STarT Back to allocate treatment to low back pain patients: Hill, Whitehurst & Lewis et al. [41], used an RCT design to compare current best practice with stratified primary care management (treatment options included advice and education, physiotherapy, and physiotherapy combined with psychological approaches) which was delivered by physiotherapists. They found that, compared with current best practice, the stratified management not only delivered improved patient disability outcomes, but also delivered cost savings. The BBPS differed from this physiotherapy-based service, providing treatment options (information, acupuncture and self-management), delivered in primary *and* community care, by experienced healthcare professionals. In addition, the BBPS was delivered in a ‘real life’ setting that used the STarT Back to recommend (as opposed to allocate) treatment options for patients. This resulted in discordance between recommended treatment options and actual treatment received for some BBPS patients (i.e. patient attendance at recommended self-management sessions was relatively poor). Nevertheless, this evaluation found no differences in pain change scores regardless of the risk of developing chronic pain. For example those at high risk of developing chronic pain improved just much as those at low risk, whereas usually poorer outcomes would be expected for those more at risk of developing chronic symptoms in non-triaged samples [47,48]. Our evaluation also found that risk of developing chronic symptoms was associated with severity of pain reported at baseline. This is in line with other studies which have also shown higher risk of chronicity to be associated with higher pain and disability scores [40]. Taken together these findings suggest that the STarT Back was a useful way to inform the triaging of this patient group and allocate resources.

Patient improvements reported by this service evaluation occurred despite high levels of pain chronicity and mental health issues among patients, which can result in poorer responses to treatment [49-51]. Anxiety and depression are common among people with chronic pain and can exacerbate pain, making them important factors to address when treating these patients [52]. BQ data showed patients in this evaluation improved on biopsychosocial dimensions of pain, including anxiety. Holistic treatment approaches such as TCM acupuncture may contribute to these improvements [24,53]. Additionally, CBT-based self-management approaches may be particularly helpful for psychosocial aspects [54,55]. The importance of self-management for back pain including psychosocial aspects is supported by findings of this service evaluation: that patients receiving self-management and acupuncture experienced greater improvements in their pain and psychosocial well-being compared with those who just received acupuncture.

Nevertheless, engaging BBPS patients in self-management was challenging. Other studies have also found that chronic pain patients may fail to follow self-management advice [56]. Despite the benefits of doing so, changing health behaviour is clearly difficult for many individuals. This may be due to a range of issues like lower socio-economic status [57]; personality traits which effect individual’s ability to make changes in their life (e.g. locus of control, self-efficacy); use of passive coping strategies such as giving responsibility of pain management to an outside source (which have been shown to predict poor outcome in back pain patients [58]); and maladaptive health beliefs and attitudes (which have been shown to influence back pain patients’ ability to engage with self-management [59]). Our findings suggest combining self-management with physical treatments which have higher attendance rates among patients, may improve access to the psychological support needed by patients most at risk of developing chronic pain.

Participants in our service evaluation reported that they particularly valued the humanistic qualities (e.g. caring, empathy) of practitioners delivering the BBPS. The importance of such qualities in healthcare professionals has been reported elsewhere [24,60,61] and is likely to be partially responsible for the current popularity of complementary and alternative medicine (CAM) [62-64]. Within a large, busy healthcare system such as the NHS these qualities can easily be side-lined by other pressing issues like outcomes and safety. Indeed, the failings at the Mid-Staffordshire NHS Foundation Trust, highlight an extreme example of how a focus on ticking boxes and meeting numerical targets can side-line patient experience, contributing to patients feeling a lack of dignity, compassion, sensitivity and care in the NHS [65]. However, in the light of this enquiry and with the Government keen that modernisation of the NHS involves putting patients “at the centre of everything the NHS does” [28] more emphasis is being placed on the quality of patient experience in the NHS. A debate is developing regarding the provision of compassionate care on the NHS [66], and measures of patient-experience are now being linked to NHS service provider pay for acute care through the CQUIN system [67]. New patient-centred models of commissioning and service redesign are also highlighting the importance of patients being heard and treated with respect [68,69].

This service evaluation is of potential interest to commissioners; firstly it demonstrates that it is possible to incorporate treatment modalities with differing underlying philosophies (i.e. Chinese acupuncture) into the NHS that are well received by patients. Secondly, although this evaluation does not compare and contrast the BBPS with other modes of CAM provision on the NHS, it does suggest that it is possible to provide CAM in a primary and community care setting, contributing to the growing body of literature that suggests that CAM can successfully be provided on the NHS in GP settings [5,24,27], special complementary therapy centres [26] or primary care centres [70]. Thirdly, this evaluation demonstrates a potential method of maximising resources through triaging patients. Finally, commissioners considering ways of putting NICE low back pain guidance into practice may also find this evaluation useful, particularly when considering ways to maximise patient participation in self-management.

### Service evaluation limitations

The current service evaluation does not report on the efficacy of the service, rather it focuses on patient outcomes and experiences of the service and some of the ‘real life’ issues involved in delivering such a service. Thus it may be useful for commissioners considering how to implement NICE low back pain guidance [1], but cannot be considered proof of efficacy of the service. A larger sample size would have provided more comprehensive data regarding the BBPS. The sample size was lower than expected due to fewer than anticipated referrals to the Service. Additionally, 26% of BBPS patients chose not to participate in the evaluation, thus the views of these non-responders are not represented by this evaluation (although there were no differences in the demographic data between responders and non-responders). However, questionnaire respondents had a varied age range, a mix of the genders and a wide variety of ethnicities (over half of our sample was from an ethnic minority), suggesting that the views of a range of respondents had been captured in the evaluation. Nevertheless, our findings should be interpreted within this context.

In addition, the evaluation only focuses on the patient experiences of the Service and not service providers or healthcare professionals involved in the Service (e.g. those able to refer to the Service). Such views and experiences would be useful in obtaining a complete picture of the usefulness of the BBPS and elucidate topics such as integrating an externally provided service into the NHS and challenges (and how they were met) with patient adherence to the self-management aspect of the programme. The Service Evaluation also did not investigate the cost implications of the Service, it is recommended that future evaluation collect such data, as this is a key interest of commissioners.

## Conclusions

The evaluation showed that the BBPS provided patients with a MSK pain management service that many found effective and valuable. The service was delivered in a primary and commuity care setting and assisted in implementing NICE Guidance for persistent low back pain locally, by working with local GPs and health professionals. Efficient BBPS triaging of patients allowed resources to be distributed appropriately according to patient need. Patients using the BBPS experienced improvements (statistically and clinically significant) in their pain, quality of life, understanding of their pain, physical activity levels and relaxation, which continued 3 months after they finished treatment (with the exception of relaxation). In addition, over one third of patients maintained a clinically significant improvement in their pain. These results are despite high levels of pain chronicity and mental health issues, which can result in poorer responses to treatment. Combining self-management with acupuncture was found to be particularly effective, although further consideration is required regarding how best to engage patients in self-management.

## Endnotes

^1^The BBPS was delivered prior to NHS reform, when PCTs were still in existence.

^2^Red flags are warning signs that indicate MSK pain may be a symptom of a more serious underlying issue and further investigations are needed (e.g. patient has history of cancer, incontinence, major trauma).

## Competing interests

The authors declare that they have no competing interests.

## Authors’ contributions

DP designed and oversaw the delivery of the BBPS, DR designed the evaluation with input from MP and AC. AC, DR and MP contributed to service evaluation implementation, and data analysis and interpretation. AC, DR and MP wrote the manuscript, DP contributed to aspects of the writing of the manuscript. All authors read and approved the final manuscript.

## Pre-publication history

The pre-publication history for this paper can be accessed here:

http://www.biomedcentral.com/1472-6882/13/300/prepub
